# The Antimicrobial Activity of Omiganan Alone and In Combination against *Candida* Isolated from Vulvovaginal Candidiasis and Bloodstream Infections

**DOI:** 10.3390/antibiotics10081001

**Published:** 2021-08-19

**Authors:** Dawid Żyrek, Andrzej Wajda, Paulina Czechowicz, Joanna Nowicka, Maciej Jaśkiewicz, Damian Neubauer, Wojciech Kamysz

**Affiliations:** 1 Department of Microbiology, Faculty of Medicine, Wrocław Medical University, 50-367 Wrocław, Poland; dawid.zyrek96@gmail.com (D.Ż.); andrzej.wajda96@gmail.com (A.W.); 2 Department of Inorganic Chemistry, Faculty of Pharmacy, Medical University of Gdańsk, 80-416 Gdańsk, Poland; mj@gumed.edu.pl (M.J.); damian.neubauer@gumed.edu.pl (D.N.); wojciech.kamysz@gumed.edu.pl (W.K.)

**Keywords:** *Candida*, biofilm, antimicrobial peptides, fluconazole, Omiganan

## Abstract

Fungi from the *Candida* genus are widespread commensals and, at the same time, are the leading cause of fungal infections worldwide. For instance, vulvovaginal candidiasis (VVC) affects approximately 75% of women at least once in their lifetime, remaining the second most common gynecological infection. On the contrary, hospital-acquired fungal bloodstream infections (BSIs), although less frequent, are characterized by a high mortality rate. Undoubtedly, the main reason for this situation are virulence factors that these yeast-like fungi can produce, and the ability to form a biofilm is one of the most important of them. Due to the low effectiveness of classic antimycotics against *Candida* biofilms, an intense search for new drugs capable of eradicating this structure is highly demanded. One of the most promising groups of compounds exhibiting such properties are antimicrobial peptides (AMPs). This study focuses on a comparison of the efficacy of Omiganan and fluconazole alone and in combination against *Candida* strains isolated from BSIs. The obtained results are consistent with our previous reports on the effectiveness of Omiganan against clinical strains isolated from VVC. This is also the first report on the combinatory application of Omiganan in the context of fungal BSI. The majority of combinations with fluconazole showed an additive effect, as well as a synergistic effect in the range of certain concentrations. Importantly, such effects are visible at concentrations much lower than for those compounds used individually. Potentially, this entails the possibility of limiting the adverse effects (e.g., toxicity) of Omiganan and fluconazole applied in vivo, thus improving the safety profile of this particular antifungal therapy.

## 1. Introduction

*Candida* species are widespread commensals that can be part of the microbiota of mucous membranes and skin. Moreover, some data show that they even occur in 50–70% of the population [1,2]. Despite the fact that *Candida* spp. belong to normal flora, they may lead to opportunistic infections and, at the same time, they are indicated as a frequent cause of symptomatic infections all over the world [3].

Vulvovaginal candidiasis (VVC) is diagnosed if the typical symptoms of vaginitis and vulvitis are accompanied by the presence of *Candida* spp. in a material obtained from the external genitalia [4]. VVC affects approximately 75% of women at least once during their lifetime, which makes it the second most common vulvovaginal infection in the world, after bacterial vaginosis (BV) [5]. In more than half of cases, VVC occurs at least twice a lifetime, and in 5–8%, at least four times a year (recurrent vulvovaginal candidiasis (RVVC)) [6]. For instance, recent epidemiological data indicate that RVVC affects nearly 138 million women annually (500 million women during their lifetime), and the number of these infections is constantly increasing. [7,8].

In fact, *Candida* spp. are noticed in every fifth healthy woman’s vaginal swabs, and the most common risk factors are pregnancy, hormone replacement therapy, diabetes, states of compromised immunity, antibiotic therapy, and steroid therapy [6,9,10]. In the majority of cases (80–95%), the etiological factor of VVC is *Candida albicans*; however, recently, the percentage of infections caused by other species belonging to this genus (non-*Candida albicans Candida* (NCAC)), such as *C. glabrata*, *C. parapsilosis, C. krusei*, and *C. tropicalis*, is increasing. In some populations, they are responsible for every fifth fungal vaginosis, occurring even more often in RVVC [6,10,11,12,13]. NCAC are usually characterized by a higher virulence and a weaker response to standard antifungal treatment, which often results in therapeutic failures [14]. Although vaginal candidiasis does not increase overall mortality, it significantly worsens the quality of life of a large number of women, causing pain, burning sensations, and discomfort, both physical and psychological, resulting in poorer self-esteem and decreased sexual satisfaction [6].

This means that VVC has become a serious and global threat, especially when considering the growing number of incidences, as well as the costs for the healthcare system. Undoubtedly, the main reason for this situation is the decreased susceptibility or resistance of *Candida* strains to azoles [15]. It should be noted that low effectiveness of the commonly used antifungals leads to prolonged therapy, which secondarily induces resistance. For *C. albicans*, several mechanisms have been described. For example, the most common are mutations in the ERG11 gene and overexpression of the efflux pumps associated genes (e.g., CDR and MDR1). The first leads to the lowered affinity of drugs, while the second prevents intracellular accumulation [16,17]. Other mechanisms include over-expression of membrane transporters and mutations in genes associated with the biosynthesis and import of sterols [18].

In bloodstream infections (BSIs), fungal cells infiltrate the systemic circulation in large numbers. As a result, they can invade internal organs and tissues, and can cause symptoms of sepsis. The most important risk factor for BSI is immunodeficiency, especially neutropenia, but other ones, such as cancer, diabetes, postoperative conditions, and treatment in the intensive care unit, cannot be ignored. Nowadays, the frequency of invasive fungal infections is increasing [19]. In fact, *C. albicans* is an etiological factor in approximately half of all cases [20,21]. However, recent data highlight that the number of infections caused by NCAC such as *C. dubliniensis, C. glabrata, C. guilliermondii, C. kefyr, C. krusei, C. parapsilosis*, and *C. tropicalis* is still rising [2,22]. Nevertheless, the multitude of virulence factors of *Candida* spp., increasing the resistance to standard antifungal drugs and the fact that BSIs mainly affect patients with comorbidities, often in profound immunosuppression, determine the poor prognosis of this group of patients [2,23,24].

Among the virulence factors of some yeast-like fungi, the ability to create a biofilm is one of the most important for their pathogenicity. It seems to be essential primarily in the case of chronic and recurrent mucocutaneous candidiasis, including RVVC, and infections related to biomaterials (artificial valves, endoprostheses, and intravascular or urinary catheters). Biofilms are characterized by a high resistance to antifungal drugs, even at very high concentrations [25,26].

Due to the low effectiveness of classic antifungals against *Candida* biofilms, an intense search for new drugs effective against this structure has arisen recently. Recent advances in this field include a quaternary ammonium derivative of pyridoxine and terbinafine with antibiofilm activity against both *Candida* and bacterial strains [27]. Studies are focused not only on derivatization, but also on the potentiation of antifungal activity of terbinafine and azoles by, e.g., the synergistic effect of 2(*5H*)-furanone derivative [28]. A relatively new and very promising group of compounds exhibiting such properties are antimicrobial peptides (AMPs), characterized by a unique mechanism of action and a relatively low potential for generating resistance [29,30].

Omiganan is a synthetic analog of indolicidin—a peptide primarily isolated from granules of bovine neutrophils [31]. Moreover, this molecule has been subjected to the final phase of clinical trials in the treatment of rosacea, juvenile acne, atopic dermatitis, genital warts, and vulvar intraepithelial neoplasia (VIN) [32,33,34,35]. The initial results also showed great potential in the treatment of fungal infections [36,37]. This study focuses on a comparison of the efficacy of Omiganan and fluconazole and their combination against planktonic forms and biofilms of blood-derived and vaginal isolates of *Candida* spp.

## 2. Materials and Methods

### 2.1. Microorganisms

The studies were conducted on clinical strains of various *Candida* species, including 32 isolates acquired from women with VVC and 30 isolates from patients with candidemia (BSI). Among the VVC strains, 30 were identified as *C. albicans*, and the other two were *C. lusitaniae* and *C. kefyr*. NCAC strains accounted for the majority (21/30) of the blood-derived isolates. These included 10 strains of *C. glabrata*, four strains of *C. parapsilosis*, three strains of *C. krusei*, two strains of *C. tropicalis*, and two strains of *C. kefyr*. The remaining nine isolates were *C. albicans* strains. All clinical strains were obtained from the internal collection of the Department of Microbiology, Wroclaw Medical University. Two reference strains (*C. albicans* ATCC 90028 and *C. glabrata* ATCC 15126) were obtained from the Polish Collection of Microorganisms (Polish Academy of Sciences, Wroclaw, Poland).

### 2.2. Peptide Synthesis

Omiganan (ILRWPWWPWRRK-NH_2_) was synthesized as described previously [38]. The peptide was synthesized manually by the solid-phase method using Fmoc chemistry on polystyrene Rink amide resin with a loading of 1.0 mmol/g (Orpegen Peptide Chemicals GmbH, Heidelberg, Germany). Single deprotection of the Fmoc group was performed with a 20% (*v*/*v*) piperidine (Merck, Darmstadt, Germany) solution in DMF (Honeywell, Seelze, Germany) for 15 min. Acylation of Fmoc-AA-OH was conducted in a DCM/DMF (1:1, *v*/*v*) solution with OxymaPure and DIC as coupling reagents (equimolar mixture of reagents) for 1.5 h using a threefold molar excess based on the resin. Every step was preceded by rinsing the resin and running the chloranil test. Cleavage of Omiganan was accomplished in TFA (Apollo Scientific, Denton, UK) and scavengers’ mixture—TIS (Sigma-Aldrich, St. Louis, MO, USA), phenol (Sigma-Aldrich, St. Louis, MO, USA), and deionized water (92.5:2.5:2.5:2.5, *v*/*v*/*v*/*v*) for 1.5 h with agitation. The cleaved peptide was precipitated with cold diethyl ether and lyophilized. Omiganan was purified by reversed-phase high-performance liquid chromatography, and its identity was confirmed by mass spectrometry (ESI-MS).

### 2.3. Antimicrobial Activity

In the first stage of the study, the minimum inhibitory concentrations (MICs) for each strain were determined. The MIC values were evaluated by the microdilution method using RPMI 1640 in accordance to the CLSI recommendations [39]. Suspensions of the tested strains (0.5–2.5 × 10^3^ CFU/mL) were applied to 96-well polystyrene plates with previously prepared serial dilutions of fluconazole or Omiganan ranging in concentration from 0.125 to 256 µg/mL. The plates were incubated for 24 h at 37 °C, and subsequently, the MICs were read. In the case of the fungistatic azole, the optical density (OD) was read spectrophotometrically (BiochromAsys UVM 340) at a wavelength of 530 nm. The MIC was considered as the concentration at which the growth inhibition of at least 50% of microorganisms was detected. For this purpose, the following equation was used: (OD_well_‒OD_K−_)/(OD_K+_‒OD_K−_) × 100%), where OD_well_ is the absorbance of the well-being assessed, OD_K−_ is the value for the negative control (background), and OD_K+_ is the value obtained in the control positive (strain growth control). With regard to the peptide, the visual method was adapted, considering the minimum inhibitory concentration value at which no turbidity, indicative of fungal growth, was observed. All experiments were conducted in triplicate and included a strain growth control (positive control) and a negative control, which served as a medium sterility test.

In the next stage, the minimum biofilm eradication concentrations (MBECs) were determined. Prepared microbial suspensions with a density of 1–5 × 10^6^ CFU/mL in RPMI 1640 medium were transferred to 96-well plates and incubated for 24 h at 37 °C. The pre-formed biofilm was then washed three times with a sterile 0.9% NaCl solution. Then, the test compounds were added to ranging concentrations of Omiganan (0.125–256 µg/mL) and fluconazole (0.25–512 µg/mL). After 24 h of incubation at 37 °C, a solution of MTT (3-(4,5-Dimethylthiazol-2-yl)-2,5-Diphenyltetrazolium Bromide) at a concentration of 1 mg/mL was added to each well and incubated for 3 h at 37 °C in the dark. Then, the results were read visually. The concentration at which no color change was observed (yellow MTT is reduced by living cells to dark colored formazan) was considered as the MBEC [40]. All experiments were conducted in triplicate and included positive and negative controls.

A preliminary assessment of the interaction between both compounds was carried out by determining the fractional inhibitory concentration index (FIC index) using the checkerboard method [41]. This assay was conducted on 24 randomly selected strains, including *C. albicans* ATCC 90028 and *C. glabrata* ATCC 15126, 10 VVC-derived strains, and 12 BSI-derived strains. The strains were numbered, and a randomization program (http://www.graphpad.com/quickcalcs/index.cfm, accessed date: 15 June 2021) was used for selection. To determine the FIC index value, 96-well plates containing serial dilutions of fluconazole (horizontal) and Omiganan (vertical) were prepared. Such an approach provided a final concentration range of 0.5–64 µg/mL for both compounds. Then, strain suspensions prepared analogically to those during the MIC determination were added to the wells of the plate. After 24 h of incubation at 37 °C, the plates were read visually, and the inhibitory concentrations were taken as those for which no growth of microorganisms was observed. The experiments were conducted in triplicate and included a positive control and a negative control. The following formulas were used to determine the FIC index:

$$\frac{A}{MIC_{A}}+\frac{B}{MIC_{B}}={\mathrm{FIC}}_{A}+{\mathrm{FIC}}_{B}=\mathrm{FIC},\;\mathrm{FIC}\;\mathrm{index}=\frac{\sum FIC}{n}$$

Compound A was fluconazole and compound B was Omiganan. The MIC concentrations previously determined for each strain were taken into account during the calculations. The values of A and B were the concentrations determined using the checkerboard method (*n* = 8). The described method is presented in Supplementary Table S1.

The FIC index values, obtained as described above, were interpreted in accordance with the recommendations [41], which are presented in Table 1.

## 3. Results

### 3.1. Minimum Inhibitory Concentration (MIC)

**Figure 1:**
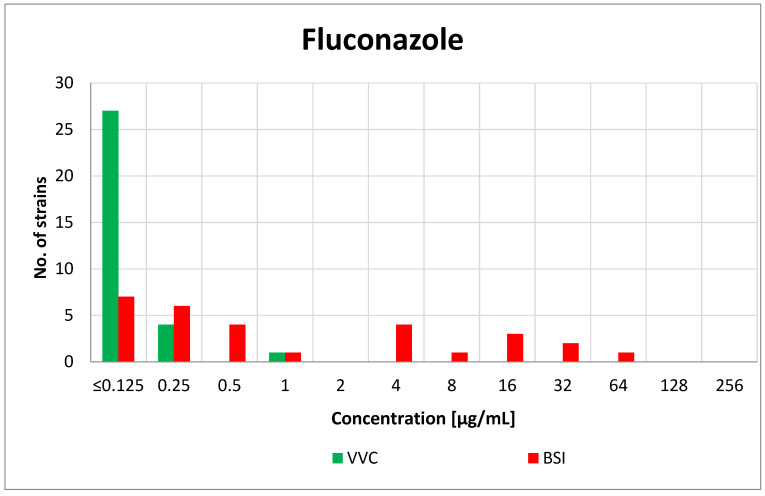
Distribution of the MICs of fluconazole among the vaginal (VVC) and blood-derived (BSI) *Candida* strains.

In the case of the VVC-derived isolates, all strains were found to be sensitive to fluconazole, and the highest MIC value was 1 µg/mL (*C. kefyr*). For the vast majority of strains (27/32, 85%) the obtained MIC was 0.125 µg/mL or less. Among the BSI-derived strains, the distribution of the MIC values was less heterogeneous and ranged from ≤0.125 to 64 µg/mL. The most common MIC was 0.125 µg/mL or less (7/30, 23%). The value of the minimum inhibitory concentration for fluconazole was not species-dependent. The MIC value distribution of fluconazole is presented in Figure 1.

**Figure 2:**
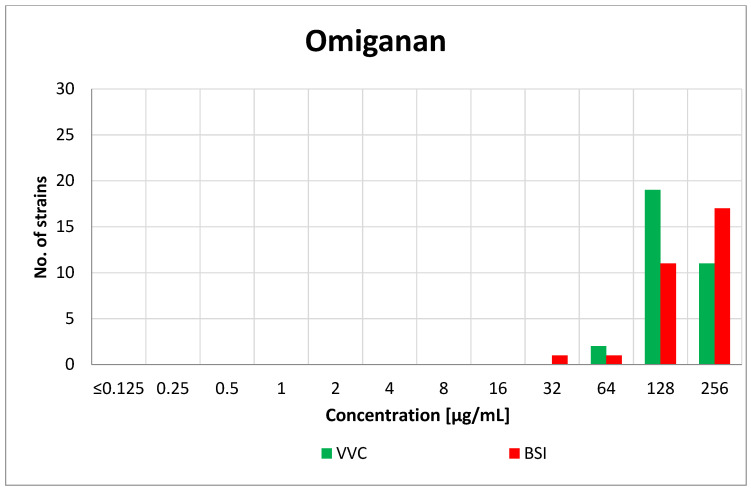
Distribution of the MICs of Omiganan among the vaginal (VVC) and blood-derived (BSI) *Candida* strains.

Omiganan turned out to be active against the tested fungi in the concentration range of 32–256 µg/mL. For the vaginal strains, the most common MIC value was 128 µg/mL (19/32, 59%). Both of the VVC-derived NCAC species presented lower MICs (64 µg/mL) than the other vaginal *C. albicans* strains. The MIC value among the blood-derived isolates was not dependent on the species, and the dominant MIC was 256 µg/mL (17/30, 57%). The MIC results for Omiganan are presented in Figure 2. Detailed data on the obtained MIC values of fluconazole and Omiganan are available in Supplementary Material in Tables S2–S4.

To determine the selectivity of Omiganan toward human and *Candida* cells, we referred to previous results from cytotoxicity studies (IC_50_) against HaCaT cell line (immortalized human keratinocytes) conducted by Jaśkiewicz et al. [42]. The IC_50_ value was included in the Selectivity Index (SI) calculation, which is its quotient and the geometric mean (GM) of the obtained MIC in this research. The results are shown in Table 2.

### 3.2. Minimal Biofilm Eradication Concentration (MBEC)

Fluconazole was ineffective in eradicating the biofilms of both the VVC and BSI-derived isolates. In both strain pools, the dominant MBEC value was 512 µg/mL (17/32, 53% for vaginal strains; 17/30, 57% for blood-derived strains). For the remaining isolates, the MBEC exceeded the applied concentration range. There were no noticeable differences in the distribution of MBECs between the VVC and BSI *Candida* isolates.

In the case of Omiganan, the MBEC values against almost all of the tested strains were equal to 256 µg/mL. Among the VVC strains, this value was observed in 97% of cases (31/32). Only one of the *C. albicans* isolates was characterized by a lower MBEC value (128 µg/mL). For all blood isolates, these values were equal to 256 µg/mL. Detailed data on the obtained MBEC values of fluconazole and Omiganan are available in Supplementary Material in Tables S2–S4.

### 3.3. FIC Index

**Figure 3:**
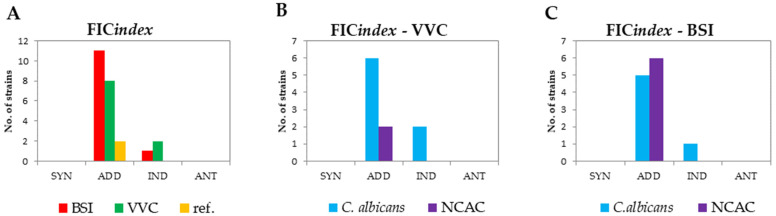
Cumulative FIC index results (checkerboard method) with the interpretation of the obtained effects of the fluconazole–Omiganan interaction. (**A**) Interpretation of the determined FIC indices with differentiation between vaginal (VVC) strains, blood-derived (BSI) isolates, and reference strains (marked as “ref.”). (**B**) Interpretation of the determined FIC indices against vaginal strains (VVC). (**C**) Interpretation of the determined FIC indices against the blood-derived strains (BSI).

The study of the interaction between fluconazole and Omiganan clearly showed an overall additive effect of the combination of these two compounds. The FIC index value for 21 out of the 24 tested strains did not exceed 1, which, according to the commonly accepted interpretation, indicates addition. For the remaining three isolates (identified as *C. albicans*) this effect was indifferent. In both the vaginal and blood-derived strains, the addition was the most common effect and affected the majority of *C. albicans* isolates (6/8 with VVC and 5/6 with BSI) and 100% of the strains from the NCAC group. The described results are shown in Figure 3.

**Figure 4:**
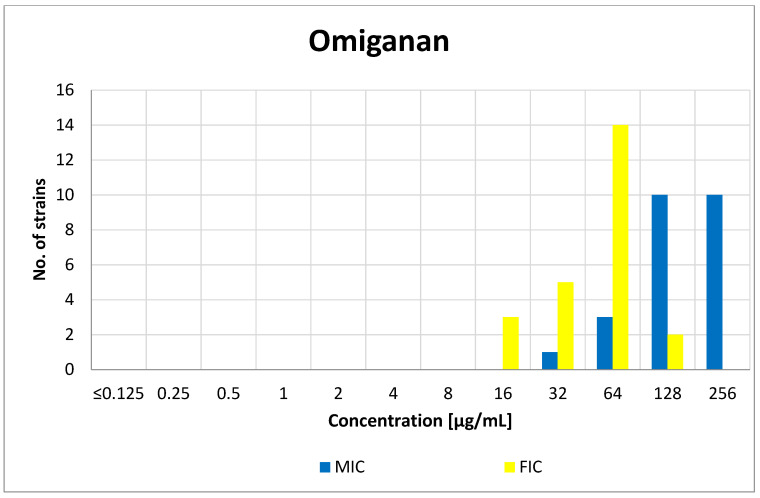
Distribution of the minimum inhibitory concentrations (MICs) of Omiganan and its concentrations at the lowest FIC that indicate synergy or addition (FIC).

As the obtained FIC indexes are the arithmetic means of eight individual FIC values calculated for various concentrations of the tested compounds, the search for the most favorable specific combination of fluconazole and Omiganan concentrations was based on the analysis of individual FICs. For each individual strain, there was a specific combination of the concentrations of fluconazole–Omiganan at which growth inhibition was observed. Taking into account such combinations and their interpretation, for 12 (out of 24) strains, it was possible to determine at least one FIC value (FIC_A_ + FIC_B_), indicating a synergistic effect (FIC ≤ 0.5). Similarly, for two out of the three strains for which an indifferent effect of the combination of fluconazole and Omiganan was observed (FIC index), it was possible to achieve an additive effect at specific compound concentrations. The peptide concentrations in the synergistic and additive combinations with fluconazole were at least one-half lower than the corresponding MICs of Omiganan alone. A comparison of the MIC values of Omiganan with the peptide concentrations indicative of the most favorable effect in combination with fluconazole for the 24 isolates tested in the checkerboard method are shown in Figure 4. The geometric mean of the MICs obtained for Omiganan was 147.89, while in case of combination of compounds, the GM of this peptide was 49.35. Detailed data on the obtained FIC values are available in Supplementary Material in Table S5.

## 4. Discussion

*Candida* species are the leading etiological factor of fungal infections worldwide [43]. The number of cases caused by these pathogens is increasing, both in the general population and among hospitalized patients [44,45]. Studies conducted so far indicate that among women of reproductive age, as many as 70–75% will experience VVC at least once in their lifetime, and almost half of them (40–50%) may experience recurrence [12,46]. To date, the most commonly isolated pathogen in women with VVC is *C. albicans*, detected in up to 90% of cases [10]. The nature of systemic candidiasis is often endogenous, and *Candida* spp. are the fourth most common trigger of infection among the patients of intensive care units. Therefore, these fungi have to be considered as a serious cause of morbidity and mortality [19,20,47,48]. *C. albicans* is responsible for more than half of all candidemias that occurs in hospitalized patients, including intensive care units and hematology departments, and 55% of all fungal infections [49,50]. Interestingly, the species belonging to the NCAC group, which are often resistant to traditional antifungal treatment, play an increasingly important role in the etiology of both VVC and BSI [10,51,52]. Numerous sources suggest that the mechanism of this resistance is multifactorial and can include incomplete penetration of antibiotics and host immune cells through the components of the extracellular matrix, initiation of structural and functional changes in biofilm cells and its environment, the ability to react to the differences in the density of the cell population by the regulation of gene expression (quorum sensing), and the expression of transport proteins that remove xenobiotics to the extracellular space (efflux pumps) [10,12,53]. Azoles are a common fungistatic drugs used in the treatment of fungal infections [54]. One of the most commonly prescribed medicaments from this group is fluconazole, often considered as the first-line drug in the prevention and treatment of yeast infections [55]. The wide range of indications and the extensive use of azoles have significantly contributed to the selection of resistant strains [54]. As a matter of fact, numerous resistance mechanisms responsible for this condition, as well as biofilm formation, are indicated [56]. Nevertheless, *Candida* spp. causing VVC clinically refractory to first-line therapy often show sensitivity to fluconazole under laboratory conditions [57,58]. It has been suggested that the key factor of the virulence responsible for the lack of therapeutic effects may be the ability to create biofilms [46,51,59]. For this reason, it is necessary to search for alternative methods of treating fungal infections based on antimicotics that effectively eradicate this structure. Antimicrobial peptides (AMPs), including Omiganan, might meet this criterion, as promising activity has already been described [38]. In the case of VVC, the MIC results obtained in this study show that fluconazole is highly active against planktonic forms of *Candida*. In 85% of cases of VVC isolates, the MIC values for fluconazole were ≤0.125 µg/mL, which is consistent with the available data from the literature [60,61]. Apart from three strains, all BSI *Candida* isolates also proved to be sensitive to fluconazole—the MIC values did not exceed 16 µg/mL. Omiganan also turned out to be active against the planktonic forms of the tested fungi from both VVC and BSI. The most common MIC values of the tested AMP were 128 µg/mL for VVC isolates and 256 µg/mL for BSI isolates. Unfortunately, taking into account previous studies on the cytotoxicity of Omiganan conducted by Jaśkiewicz et al., attention should be paid to the poor selectivity of this compound toward *Candida*, since the calculated selectivity index appeared to be very low (0.48) [42]. However, to assess the safety profile reliably, comprehensive research on its cytotoxicity—for example, toward the vaginal epithelium cell lines—should be conducted. Based on the obtained very high biofilm eradication concentrations of fluconazole, it should be concluded that this mycostatic compound is ineffective against fungal biofilm, which has already been emphasized in the literature [62,63]. On the contrary, the obtained MBECs of Omiganan were also relatively high for the majority of strains (256 µg/mL) (61/62). However, the activity of this compound against planktonic forms (MIC) was identical or similar to the activity against *Candida* biofilms (MBEC). In the case of 44% (28/64) of the strains, the MIC value was identical to the MBEC. For another 47% (30/64), these values differed only by one order of magnitude. Considering the need to conduct more detailed studies on the toxicity of Omiganan, its greatest advantage may be the comparable activity against *Candida* in both the planktonic forms and biofilms.

The FIC indexes representing the simultaneous use of both tested drugs turned out to be particularly interesting. Although, initially, in our previous report for three strains of *Candida* spp., the FIC determination indicated an antagonism between Omiganan and fluconazole. However, in the course of our studies on a larger group of strains, we found an additive and/or synergistic effect [38]. In both the vaginal and blood-derived strains, addition was the most common effect, affecting the majority of *C. albicans* isolates (6/8 with VVC and 5/6 with BSI) and 100% of the strains from the NCAC group. Among the strains that showed synergy and/or addition in the action of Omiganan and fluconazole, the MIC values for each of these substances used alone were at least two times higher than those obtained in combination. The observed outcomes demonstrate the potential of combined therapy with the use of AMPs and classic fungistatics. The likely reason for the greater effectiveness of the combination of both compounds is the complementarity of their mechanisms of action. After penetrating into the fungal cell, fluconazole inhibits the Erg11 enzyme involved in the synthesis of ergosterol, being a component of the fungal cell membrane. It has been observed that under the influence of azoles, there is a change in the fungal cell membrane correlated with the disturbance of its structure and increased permeability to ATP and K^+^ ions, which is directly responsible for the fungistatic effect of this group of compounds [2,64,65]. Omiganan, on the contrary, acts directly on the fungal cell membranes, leading to their permeabilization and, as a result, cell death [38]. It is assumed that Omiganan facilitates the penetration of fluconazole into the cell interior, and thus helps to achieve its molecular target [33,66]. Similar observations indicating a positive effect of the combined action of fluconazole and AMPs have been observed in many other studies [38,67,68,69,70]. For example, AMPs showed synergistic activity against *C. albicans* not only when used with fluconazole, but also with capsofungin—a semi-synthetic lipopeptide [70]. Almost identical results concerning the combination therapy of Omiganan and fluconazole were obtained with the use of retro-Omiganan [38]. In the case of fungal strains resistant to standard treatment, it seems reasonable to search for alternative forms of therapy by examining the effect of combination therapy. Such an approach may not only allow for more effective treatment of severe or recurrent fungal infections, but it will also presumably lead to a reduction of the dose of the used antifungal drug, hence lowering the risk of complications of pharmacotherapy.

## 5. Conclusions

Our present study on BSI isolates confirmed previous reports on the effectiveness of Omiganan as a biocide against *Candida* spp. clinical strains, particularly in biofilm forms, isolated from the vaginas of women with vulvovaginal candidiasis. Despite the observed strong antifungal properties of Omiganan, its advantage over fluconazole in inhibiting the growth of the planktonic forms of *Candida* was not demonstrated, but regarding the biofilms, it was still more effective than conventionally used azole. The combined use of both compounds generally showed an additive effect, but at some concentrations, they acted synergistically. The described outcome occurred at concentrations well below the MIC values for fluconazole or Omiganan when they are tested separately. Such a result indicates the possibility of reducing their concentrations in combination therapy, thus lowering the risk of side effects using combination therapy. Nevertheless, due to the limited number of tested strains and the incomplete data on the toxicity of synthetic antimicrobial peptides, especially in combination with other drugs, it is necessary to conduct further research in this area.

## Figures and Tables

**Table 1:** Interpretation of the interactions between the two compounds tested using the checkerboard method, based on of the obtained FIC index values.

FIC Index	Interpretation
≤0.5	Synergism (SYN)
>0.5 to 1.0	Addition (ADD)
>1.0 to ≤2.0	Indifference (IND)
>2.0	Antagonism (ANT)

**Table 2:** Geometric mean of the MIC values (GM), IC_50_, and calculated Selectivity Index (SI) of Omiganan against combined vaginal (VVC) and blood-derived (BSI) *Candida* strains.

Peptide	GM	IC_50_ [42]	SI
Omiganan	166.43	79.39	0.48

## Data Availability

All data generated or analyzed during this study are included in this published article and supplementary files.

## Supplementary Materials

The following are available online at https://www.mdpi.com/article/10.3390/antibiotics10081001/s1. Table S1. The checkerboard method. The blue color indicates fluconazole concentrations, while the green color indicates Omiganan concentrations; both cases within the range of 0.5–64 µg/mL. K+ is the positive control (microorganism growth control), and K- is the medium sterility control (negative control). Table S2. MIC and MBEC values (µg/mL) of fluconazole and Omiganan against reference *Candida* strains. Table S3. Distribution of MIC and MBEC values (µg/mL) of fluconazole and Omiganan among vaginal (VVC) *Candida* strains. Table S4. Distribution of MIC and MBEC values (µg/mL) of fluconazole and Omiganan among vaginal (VVC) and blood-derived (BSI) *Candida* strains. Table S5. Correlation between the lowest obtained FIC values and the corresponding concentration (µg/mL) of Omiganan, compared to the MIC (µg/mL) of Omiganan against 24 random selected *Candida* isolates.
