# Supplementary material for: The Antimicrobial Activity of Omiganan Alone and In Combination against Candida Isolated from Vulvovaginal Candidiasis and Bloodstream Infections
Source: Antibiotics (Basel). 2021 Aug 19;10(8):1001. doi: 10.3390/antibiotics10081001 (PMC8389017; doi:10.3390/antibiotics10081001)
Supplement: Supplementary file 1 [file antibiotics-10-01001-s001.zip › antibiotics-1335029-supplementary.pdf]

antibiotics

Article – Supplementary materials

The Antimicrobial Activity of Omiganan Alone and In Combination against *Candida* Isolated from Vulvovaginal Candidiasis and Bloodstream Infections

Dawid Żyrek [1], Andrzej Wajda [1], Paulina Czechowicz [1,*], Joanna Nowicka [1,*], Maciej Jaśkiewicz [2], Damian Neubauer [2] and Wojciech Kamysz [2]

1 Department of Microbiology, Faculty of Medicine, Wrocław Medical University; 50-367 Wrocław, Poland; dawid.zyrek96@gmail.com (D.Ż.); andrzej.wajda96@gmail.com (A.W.)
2 Department of Inorganic Chemistry, Faculty of Pharmacy, Medical University of Gdańsk, 80-416 Gdańsk, Poland; mj@gumed.edu.pl (M.J.); damian.neubauer@gumed.edu.pl (D.N.); wojciech.kamysz@gumed.edu.pl (W.K.)
* Correspondence: paulina.czechowicz.umedwroc@gmail.com (P.C.); joanna.nowicka@umed.wroc.pl (J.N.)

Table S1. The checkerboard method. Blue color indicates fluconazole concentrations, and green color indicates Omiganan concentrations, in both cases within the range of 0.5–64 µg/mL. K+ is the positive control (microorganism growth control), and K– is the medium sterility control (negative control).

No of row	1	2	3	4	5	6	7	8	9	10	11	12
A	64	32	16	8	4	2	1	0.5	K+	K–		
	64	64	64	64	64	64	64	64				
B	64	32	16	8	4	2	1	0.5	K+	K–		
	32	32	32	32	32	32	32	32				
C	64	32	16	8	4	2	1	0.5	K+	K–		
	16	16	16	16	16	16	16	16				
D	64	32	16	8	4	2	1	0.5	K+	K–		
	8	8	8	8	8	8	8	8				
E	64	32	16	8	4	2	1	0.5	K+	K–		
	4	4	4	4	4	4	4	4				
F	64	32	16	8	4	2	1	0.5	K+	K–		
	2	2	2	2	2	2	2	2				
G	64	32	16	8	4	2	1	0.5	K+	K–		
	1	1	1	1	1	1	1	1				
H	64	32	16	8	4	2	1	0.5	K+	K–		
	0.5	0.5	0.5	0.5	0.5	0.5	0.5	0.5				

Table S2. MIC and MBEC values [µg/mL] of fluconazole and Omiganan against reference *Candida* strains.

Strain	Fluconazole		Omiganan	
	MIC	MBEC	MIC	MBEC
C. albicans ATCC 90028	0.125	1024	256	256
C. glabrata ATCC 15126	4	1024	128	256

Table S3. Distribution of MIC and MBEC values [µg/mL] of fluconazole and Omiganan among vaginal (VVC) *Candida* strains.

No	Strain	Fluconazole		Omiganan	
		MIC	MBEC	MIC	MBEC
V1	*C. albicans*	0.125	512	128	128
V2	*C. albicans*	0.125	512	256	256
V3	*C. albicans*	0.125	1024	256	256
V4	*C. albicans*	0.125	1024	256	256
V5	*C. lusitaniae*	0.25	1024	64	256
V6	*C. kefyr*	1	512	64	256
V7	*C. albicans*	0.125	1024	256	256
V8	*C. albicans*	0.125	1024	128	256
V9	*C. albicans*	0.125	1024	128	256
V10	*C. albicans*	0.125	1024	128	256
V11	*C. albicans*	0.125	1024	128	256
V12	*C. albicans*	0.125	1024	256	256
V13	*C. albicans*	0.125	512	128	256
V14	*C. albicans*	0.125	1024	128	256
V15	*C. albicans*	0.25	512	128	256
V16	*C. albicans*	0.125	1024	128	256
V17	*C. albicans*	0.125	512	128	256
V18	*C. albicans*	0.125	1024	256	256
V19	*C. albicans*	0.125	512	256	256
V20	*C. albicans*	0.125	512	128	256
V21	*C. albicans*	0.125	512	256	256
V22	*C. albicans*	0.125	512	256	256
V23	*C. albicans*	0.125	512	256	256
V24	*C. albicans*	0.125	512	128	256
V25	*C. albicans*	0.125	512	128	256
V26	*C. albicans*	0.125	512	128	256
V27	*C. albicans*	0.125	1024	128	256
V28	*C. albicans*	0.125	1024	256	256
V29	*C. albicans*	0.125	512	128	256
V30	*C. albicans*	0.25	512	128	256
V31	*C. albicans*	0.125	512	128	256
V32	*C. albicans*	0.25	1024	128	256

Table S4. Distribution of MIC and MBEC values [µg/mL] of fluconazole and Omiganan among vaginal (VVC) and blood-derived (BSI) *Candida* strains.

No	Strain	Fluconazole		Omiganan	
		MIC	MBEC	MIC	MBEC
B1	*C. kefyr*	4	512	256	256
B2	*C. parapsilosis*	0.5	1024	256	256
B3	*C. glabrata*	0.125	512	128	256
B4	*C. parapsilosis*	16	512	256	256
B5	*C. parapsilosis*	0.5	512	64	256
B6	*C. glabrata*	4	512	256	256
B7	*C. albicans*	0.125	512	256	256
B8	*C. glabrata*	4	512	256	256
B9	*C. tropicalis*	0.25	1024	32	256
B10	*C. tropicalis*	0.25	512	128	256
B11	*C. parapsilosis*	0.25	1024	256	256

B12	*C. krusei*	32	1024	128	256
B13	*C. albicans*	16	1024	128	256
B14	*C. glabrata*	8	512	256	256
B15	*C. albicans*	0.25	1024	128	256
B16	*C. glabrata*	0.5	512	128	256
B17	*C. glabrata*	0.5	1024	256	256
B18	*C. glabrata*	64	512	256	256
B19	*C. albicans*	0.25	1024	256	256
B20	*C. krusei*	32	512	128	256
B21	*C. glabrata*	0.25	512	256	256
B22	*C. albicans*	0.125	512	128	256
B23	*C. albicans*	0.125	1024	128	256
B24	*C. krusei*	16	1024	128	256
B25	*C. albicans*	0.125	1024	256	256
B26	*C. albicans*	0.125	1024	256	256
B27	*C. glabrata*	0.25	1024	256	256
B28	*C. glabrata*	4	512	256	256
B29	*C. kefyr*	0.125	512	128	256
B30	*C. glabrata*	1	512	256	256

Table S5. Correlation between the lowest obtained FIC values and corresponding concentration [μg/mL] of Omiganan, compared to MIC [μg/mL] of Omiganan against 24 random selected *Candida* isolates.

No	isolation	Strain	The lowest FIC	Interpretation of FIC*	Concentration of Omiganan Corresponding to FIC	MIC of Omiganan
-	*C. albicans* ATCC 90028		0.266	S	64	256
-	*C. glabrata* ATCC 15126		0.266	S	32	128
B9	BSI	*C. tropicalis*	0.515	A	16	32
B5	BSI	*C. parapsilosis*	0.531	A	32	64
B3	BSI	*C. glabrata*	0.515	A	64	128
B17	BSI	*C. glabrata*	0.5	S	32	128
B23	BSI	*C. albicans*	0.515	A	64	128
B24	BSI	*C. albicans*	0.563	A	64	128
B7	BSI	*C. albicans*	0.266	S	64	256
B22	BSI	*C. glabrata*	0.141	S	32	256
B27	BSI	*C. albicans*	0.266	S	64	256
B28	BSI	*C. albicans*	0.515	A	128	256
B13	BSI	*C. albicans*	0.141	S	16	128
B14	BSI	*C. glabrata*	0.5	S	64	256
V5	VVC	*C. lusitaniae*	0.281	S	16	64
V6	VVC	*C. kefyr*	0.625	A	32	64
V8	VVC	*C. albicans*	1.016	I	128	128
V13	VVC	*C. albicans*	0.515	A	64	128
V14	VVC	*C. albicans*	0.563	A	64	128
V16	VVC	*C. albicans*	0.515	A	64	128
V2	VVC	*C. albicans*	0.266	S	64	256
V3	VVC	*C. albicans*	0.266	S	64	256
V4	VVC	*C. albicans*	0.266	S	64	256
V7	VVC	*C. albicans*	0.266	S	64	256

*S-synergy; A-Additive, I- Indifferent.
